# Biliary and portal vein strictures following treatment of Hodgkin’s lymphoma

**DOI:** 10.1308/003588412X13373405385854

**Published:** 2012-10

**Authors:** KJ Roberts, R Brown, JV Patel, GJ Toogood

**Affiliations:** Leeds Teaching Hospitals NHS Trust,UK

**Keywords:** Jaundice, Portal vein, Lymphoma, Interventional radiology

## Abstract

Treatment of abdominal lymphoma can be associated with bowel stricture or perforation. Rarely, the common bile duct or portal vein can be involved. This is the first case of stricture formation of both the portal vein and common bile duct in a patient following successful treatment of lymphoma. The development of extensive hilar varices rendered surgical management high risk. A staged approach to treatment was used. First, a percutaneous portal vein stent was placed, resulting in resolution of the hilar varices. This was followed by a surgical hepaticojejunostomy, performed without complication. Gastrointestinal complications are rare following treatment of lymphoma but may affect a variety of sites. The safe and effective treatment of this case highlights the benefit of a multidisciplinary approach to complex medical and surgical problems.

Gastrointestinal complications can follow treatment of abdominal lymphoma due to tumour necrosis and include bowel perforation or stricture formation. This is the first report of combined common bile duct (CBD) and portal vein (PV) stricture following treatment of abdominal lymphoma. Extensive hilar varices, as a result of portal venous occlusion, prevented initial safe surgical treatment. A staged multi-disciplinary approach consisting of radiological intervention followed by surgery led to a successful and uncomplicated outcome.

## Case history

**Figure 1:**
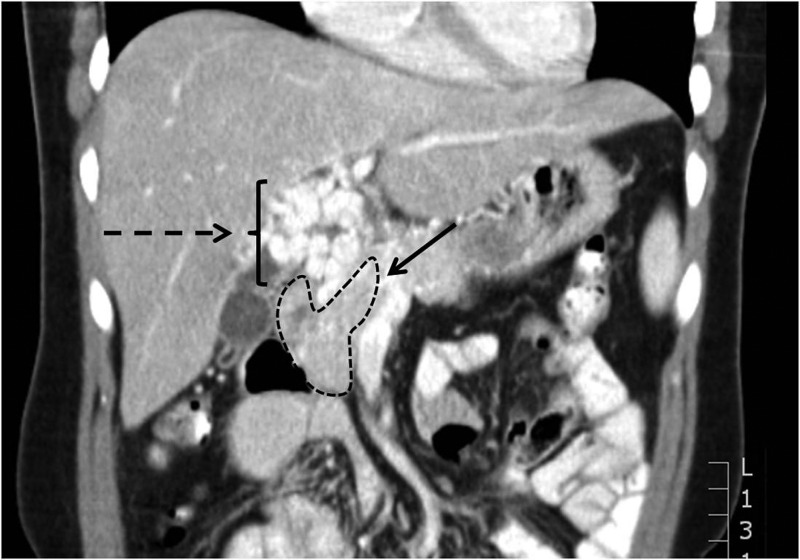
The mass is demonstrated in the hatched area and seen to cause obstruction of the portal vein (solid arrow). Numerous varices are seen (dashed arrow) that reform the intrahepatic portal vein.

A 28-year-old woman presented with a 2-week history of abdominal discomfort. Clinical examination revealed jaundice but was otherwise unremarkable. Imaging demonstrated a retroperitoneal mass (6cm × 5cm) at the liver hilum (Fig 1). The main PV was occluded with extensive upper abdominal varices. The distal extrahepatic bile duct was obstructed with intrahepatic biliary dilatation, treated by endoscopic placement of a plastic biliary stent. A diagnosis of nodular sclerosing Hodgkin’s lymphoma was made after ultrasonography guided biopsy demonstrated a dense lymphoid infiltrate, predominantly of T cells with classical Reed–Sternberg cells in a fibrous stroma. The patient completed a full course of chemotherapy (doxorubicin, bleomycin, vinblastine, dacarbazine) with excellent response. Positron emission tomography demonstrated no residual disease. With tumour resolution, the biliary obstruction resolved. At this point, the biliary stent was removed with cholangiography demonstrating no CBD stricture.

**Figure 2:**
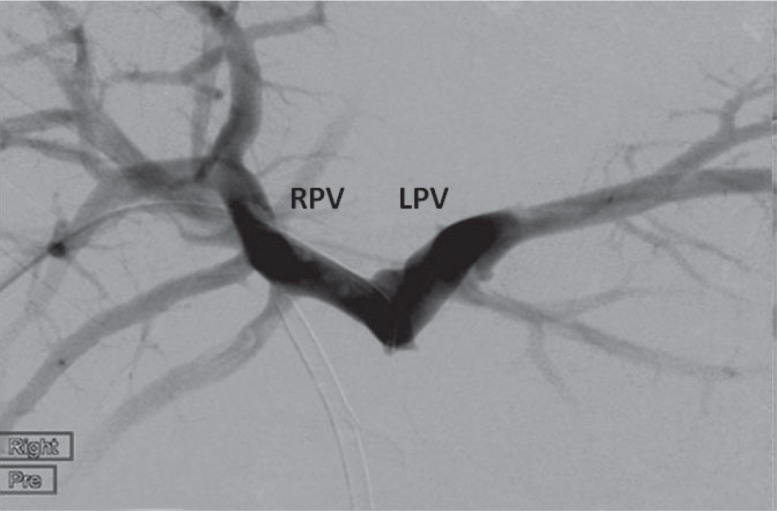
Pre-stent venography demonstrated an occluded main portal vein with filling of the right and left portal veins (RPV and LPV).

The patient re-presented one year later with jaundice. Cholangiography demonstrated a smooth stricture of the distal CBD. Computed tomography (CT) demonstrated no evidence of disease recurrence but highlighted stenosis of the main PV with cavernous reformation and the presence of large varices (Fig 2). She was referred for bile duct reconstruction but at a multidisciplinary forum it was agreed that this would be difficult due to the extensive hilar varices. Therefore, PV stenting was planned in order to decompress the varices prior to surgery.

**Figure 3:**
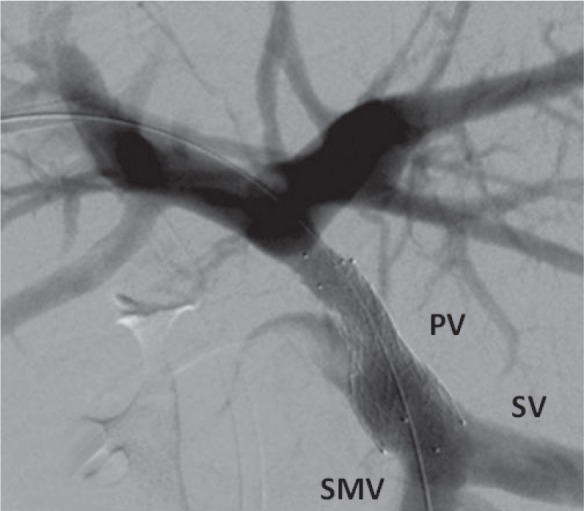
Expanded stent in main portal vein (PV) inserted over a guidewire. The PV filled via the patent splenic and superior mesenteric veins (SV and SMV).

PV stent insertion was performed via a percutaneous transhepatic approach. The occlusion was stented with self-expanding stents (Protégé® GPS™, eV3 Endovascular, Plymouth, MN, US) (Fig 3). Subsequent CT demonstrated resolution of the hilar varices. Subsequently, an elective Roux-en-Y hepaticojejunostomy was performed. Dense scar tissue surrounding the CBD was encountered but there were no varices. The patient made an uneventful recovery with no complications and remains well 12 months after surgery.

## Discussion

Lymphoma very rarely involves the CBD.^1^ This is the first case of combined PV and CBD stricture following lymphoma treatment. Chemotherapy is curative in the majority of cases of gastrointestinal lymphoma resulting in tumour necrosis. Presumably if a significant proportion of bowel wall or bile duct is involved, stricture formation or perforation may occur.^2^ Stricture of the CBD following chemotherapy for lymphoma has been reported previously^1^ as have strictures of the oesophagus, stomach and small bowel. If significant organ involvement is demonstrated on cross-sectional imaging prior to chemotherapy, the potential for these complications can be predicted.

The management of the CBD stricture was complicated by the development of portal hypertension and varices secondary to PV occlusion. Surgical treatment in the form of portosystemic anastomosis was an option but this is associated with significant morbidity and long-term risk of encephalopathy from a non-physiological shunt. Its role is limited due to advances in percutaneous endovascular techniques. Transhepatic PV stenting has been described widely to treat strictures following liver transplantation^3^ and, occasionally, as palliation^4^ or as part of curative treatment^5^ for hepatopancreatobiliary malignant disease. The use of PV stenting in the present case permitted resolution of portal hypertension and decreased the risk associated with performing surgical hepaticojejunostomy, a very difficult operation in the presence of hilar varices.

## Conclusions

A multidisciplinary approach was key to the successful management of this case highlighting the importance of close working relationships between surgeons and interventional radiologists.
